# Sero-prevalence and vaccination status of hepatitis A and hepatitis B among adults with cirrhosis in Sri Lanka: a hospital based cohort study

**DOI:** 10.1186/s13104-017-2634-5

**Published:** 2017-07-21

**Authors:** Madunil Anuk Niriella, Vipuli Jayendra Kobbegala, Hasnatha Nuwan Karalliyadda, Chamila Kumara Ranawaka, Arjuna Priyadarshin de Silva, Anuradha Supun Dassanayake, Hithanadura Janaka de Silva

**Affiliations:** 1 Department of Medicine, Faculty of Medicine, University of Kelaniya, PO Box 6, Thalagolla Road, Ragama, 11010 Sri Lanka; 2 Peripheral General Hospital, Rathnapura, Sri Lanka

**Keywords:** Hepatitis A, Hepatitis B, Immunity, Cirrhosis, Sri Lanka

## Abstract

**Background:**

As acute viral hepatitis can be fatal in patients with cirrhosis, vaccination against hepatitis A (HAV) and hepatitis B (HBV) is recommended for non-immune patients. With increasing affluence the incidence of hepatitis A in childhood has decreased leading to a significant proportion of non-immune adults. As part of their routine investigation, hepatitis A IgG antibodies (anti-HAV IgG), hepatitis B surface antigen (HBsAg) and anti-HCV antibodies was checked and immunization status was assessed among consenting newly diagnosed cirrhotic patients presenting to a tertiary referral center.

**Findings:**

Out of 135 patients, 107 [79.3%; males 91; mean age (SD) at presentation: 55.5 (11.6) years] with complete data were included for analysis. Most patients had either cryptogenic cirrhosis (62.6%) or alcoholic cirrhosis (29.9%); 2 (1.9%) had HBV cirrhosis, none had hepatitis C (HCV) cirrhosis. None of the patients had received vaccination against hepatitis A, while 71 (67.6%) had been vaccinated against HBV. The majority [62 (58%)] were negative for anti-HAV IgG.

**Conclusion:**

Most cirrhotic patients in this cohort were not immune to hepatitis A. None had been vaccinated against HAV, while a third of patients had not been vaccinated against HBV. Cirrhotic patients should be routinely investigated for immunity against HAV and HBV, and vaccination offered to those found to be non-immune.

## Findings

### Introduction

Hepatitis A virus (HAV) is a common, often asymptomatic infection in childhood in developing countries [1]. Due to improvements in sanitation and hygiene, the incidence of childhood HAV has decreased in many developing middle-income countries such as Sri Lanka, leading to a significant proportion of non-immune adults in the community [1]. Both HAV and hepatitis B virus (HBV) can cause severe infection in non-immune adults, and can even be fatal in adult patients with cirrhosis. Vaccination against HAV and HBV is, therefore, recommended for non-immune patients with cirrhosis [2].

There is limited data on HAV and HBV vaccination among Sri Lankan patients with cirrhosis. The objective of this study was to investigate the sero-prevalence and vaccination status for HAV and HBV in a cohort of adult patients with cirrhosis.

### Methods

The study was conducted in the Gastroenterology out-patient clinic of the University Medical Unit, Colombo North Teaching Hospital, Ragama during 1 year period from January 2013 to 2014. Consecutive, consenting, newly diagnosed patients with cirrhosis with at least 3 months follow up were included. As part of their routine investigations, Hepatitis A Ig G antibody (anti-HAV IgG), hepatitis B surface antigen (HBsAg) and anti-hepatitis C virus (HCV) antibodies were checked using CTK BIOTECH ELISA kits. Demographic data, possible aetiology of cirrhosis, and HAV and HBV immunization status as documented in the case records, were recorded. Ethical approval for the study was obtained from the Ethical Review Committee of the Faculty of Medicine, University of Kelaniya.

### Results

Out of 135 patients, 107 [79.3%] with complete data were included in the analysis. There were 91 (85%) males and mean age (SD) at presentation was 55.8 (11.6) years. Most patients had either cryptogenic cirrhosis (62.6%) or alcoholic cirrhosis (29.9%). Only 2 (1.9%) had HBsAg positive HBV cirrhosis, while there was no HCV cirrhosis. 45 (42%) patients were positive and 62 (58%) were negative for anti-HAV IgG. None of the patients had received vaccination against hepatitis A while 71 (67.6%) patients had been vaccinated against HBV.

### Discussion

Hepatitis A is endemic in Sri Lanka [1]. Therefore, it was expected that a majority of patients will have immunity to HAV through past infection. Contrary to this we found that nearly 60% of the adult cirrhotic patient were non-immune to HAV. None had been vaccinated for HAV even after 3 months of follow up. This leaves a high proportion of cirrhotic adults with no immunity to HAV and, therefore, at risk of a potentially fatal HAV infection.

There are few published data on sero-prevalence of HAV among Sri Lankans. Moritsugu et al. [3] reported a 76.9% sero-prevalence among healthy individuals from Colombo in 1979. In 2005, de Silva et al. reported a 10.8% sero-prevalence among children attending a tertiary referral hospital [4], reflecting a decreasing incidence of the infection due to improved hygiene and sanitation. Only 42% of cirrhotic patients in the present study were immune to Hepatitis A. This further reflects improved sanitation and general hygiene with development resulting in reduced transmission of feco-oral infection such as HAV in countries such as Sri Lanka.

The very low rates of HBV (<2%) and HCV cirrhosis (<1%) observed in this study, is in keeping with previously published studies from Sri Lanka [5]. Only a small minority of our patients had HBV related cirrhosis, and two-thirds of them had been vaccinated against HBV. Although suboptimal, HBV immunization seems to be a relatively established practice, while HAV immunization is neglected.

We were not able to identify the contributory factors for the low level of immunization against HAV among our patients. A false perception of high prevalence of acquired natural immunity to HAV among patients with cirrhosis by doctors may have contributed to the lack of immunization for HAV. Furthermore, non-availability of HAV vaccine, free of charge in the state sector, where the study was conducted, would have contributed the absence of immunization among this cohort. Conversely, HBV vaccine is freely available in the state sector. This would have contributed to two-thirds of the cohort being vaccinated for HBV. Additionally, we were not able to check for immunity against HBV as there was no testing for anti-Hepatitis B core IgG antibody (anti-HBc Ab) among the HBsAg positive patients and levels of anti-Hepatitis B surface antibody (anti-HBs Ab) among the vaccinated patients.

In conclusion, there was absence of HAV and sub-optimal HBV vaccination in this cohort of cirrhotics, especially among non-immune patients. Given that both HAV and HBV can cause potentially fatal infections in adult patients with cirrhosis, immune status for both infections should be routinely checked in newly diagnosed Sri Lankan patients with cirrhosis. Immunization should be offered to all those found to be non-immune to prevent life threatening future infections. The above findings may be applicable to countries in which HAV is endemic, where past HAV exposure is may be not as high as expected, due to similar improvements socio-economic status as in Sri Lanka. In these countries screening for patients with chronic liver disease for hepatitis A exposure with anti-HAV IgG antibodies and if negative vaccinating them will save lives by preventing potentially fatal acute hepatitis among patients with established cirrhosis.

## Abbreviations

HAV: hepatitis A virus
HBV: hepatitis B virus
Anti-HAV IgG: hepatitis A IgG antibody
HBsAg: hepatitis B surface antigen
HCV: hepatitis C virus
Anti-HBc Ab: anti-hepatitis B core IgG antibody
Anti-HBs Ab: anti-hepatitis B surface antibody
